# Determination of crAssphage in water samples and applicability for tracking human faecal pollution

**DOI:** 10.1111/1751-7915.12841

**Published:** 2017-09-19

**Authors:** Cristina García‐Aljaro, Elisenda Ballesté, Maite Muniesa, Juan Jofre

**Affiliations:** ^1^ Department of Genetics, Microbiology and Statistics University of Barcelona Diagonal 643 08028 Barcelona Spain

## Abstract

In recent decades, considerable effort has been devoted to finding microbial source‐tracking (MST) markers that are suitable to assess the health risks of faecally polluted waters, with no universal marker reported so far. In this study, the abundance and prevalence of a crAssphage‐derived DNA marker in wastewaters of human and animal origins were studied by a new qPCR assay with the ultimate aim of assessing its potential as an MST marker. crAssphage showed up to 10^6^ GC/ml in the sewage samples of human origin, in both the total DNA and the viral DNA fraction. In wastewaters containing animal faecal remains, 39% of the samples were negative for the presence of the crAssphage sequence, while those showing positive results (41% of the samples) were at least 1 log_10_ unit lower than the samples of human origin. Noteworthy, the log_10_ values of the ratio (R) crAssphage (GC/ml)/*Escherichia coli* (CFU/ml) varied significantly depending on the human or animal origin (*R* > 1.5 for human samples and *R* < −1.5 for animal wastewater samples. This study opens the way for further research to explore if different specific animal variants of crAssphage exist and whether other zones of the crAssphage genome are better suited to source discrimination.

## Introduction

Waterborne diseases transmitted via the faecal–oral route make a significant contribution to the burden of diseases worldwide (WHO, 2008). Faecal pollution has several origins, human or diverse animals, and determination of the faecal sources accompanied by appropriate water resource management policies could contribute to improving the microbial quality of water (Blanch *et al*., 2006; Hagedorn *et al*., 2011). Source‐tracking methods allow the origin of faecal pollution in a particular body of water to be determined (Scott *et al*., 2002; U.S. EPA, 2005; Hagedorn *et al*., 2011; Harwood *et al*., 2014). A number of approaches have been developed to identify the source of faecal pollution, in attempts to associate various animals with faecal contamination of natural waters (Blanch *et al*., 2006; Harwood *et al*., 2014). These methods are in various stages of development and approval. Those based on microbiological determination are defined as microbial source‐tracking (MST) methods.

The analysis of gastrointestinal microorganisms indicates that about 400 different species of bacteria can be found in the animal intestine and that populations are of the order of 10^11^/g, although only a very small fraction of these bacteria have been cultured (Zoetendal *et al*., 2004). The intestinal microbiome has been characterized in detail in several animal hosts, including humans (Eckburg *et al*., 2005; Gill *et al*., 2006), pigs (Leser *et al*., 2002) and cattle (Ramsak *et al*., 2000). Culture‐independent methods suggest that numerically dominant bacteria in the colon of animals are anaerobic and belong to the bacterial phylum Cytophaga‐Flavobacter‐Bacteroides, with *Bacteroides* being one of the most common genera in the animal intestine (Matsuki *et al*., 2002; Eckburg *et al*., 2005; Ley *et al*., 2008; Bradford *et al*., 2013; Ishikawa *et al*., 2013; Wei *et al*., 2013). However, anaerobic bacteria (*Bacteroides*,* Bifidobacterium*) are not easily grown in the laboratory, which has limited their use as faecal indicators in the past, and for this reason molecular methods for their detection have been developed (Bernhard and Field, 2000; Haugland *et al*., 2010; Gómez‐Doñate *et al*., 2012; Green *et al*., 2014; Mayer *et al*., 2016).

In this context, the molecular detection of host‐specific *Bacteroides* species as well as bacteriophages infecting different *Bacteroides* species (Tartera and Jofre, 1987; Payan *et al*., 2005; Gómez‐Doñate *et al*., 2011; Jofre *et al*., 2014; McMinn *et al*., 2014; Venegas *et al*., 2015) have proved to be useful in MST studies. However, a drawback with both approaches is a certain geographical inconsistency of the results (Reischer *et al*., 2013; Jofre *et al*., 2014).

Recent metagenomic studies of human intestinal contents, and ‘*in silico’* analyses have revealed DNA sequences present in the majority of published human faecal metagenomes (Dutilh *et al*., 2014). These sequences have been identified as belonging to a single, previously unidentified bacteriophage, named crAssphage after the *cross assembly* program used to generate it (Dutilh *et al*., 2014). This virtual bacteriophage was assigned to *Bacteroides* ssp in accordance with CRISPR analysis. However, the phage does not resemble previously reported *Bacteroides* phages like phage B‐40 (ATCC 51477‐B1 eB40‐8) infecting *Bacteroides fragilis* (Tartera and Jofre, 1987) and phage ΦB124‐14 which infects strain GB‐124 of *B. fragilis* (Ogilvie *et al*., 2012).

This study presents the molecular detection of a crAssphage‐derived DNA sequence (referred to herein as crAssphage) in samples with faecal pollution of known origin (human or animal) using a newly designed qPCR and evaluates the differential occurrence of crAssphage in human‐polluted samples and its potential as a molecular MST marker.

## Results and discussion

### Sequencing crAssphage amplimer from HM samples

In order to design a qPCR to detect the crAssphage genome first, a total of 10 samples of human (HM) sewage DNA samples were amplified and sequenced using primers reported in Fig. S1 targeting a region of 1331 bp. The amplicons generated revealed certain sequence variation, indicating that different crAssphage variants may exist. This is in accordance with previous results from Liang and coworkers that showed high genetic diversity in ORF00018 and ORF00039 of the crAssphage sequence available at GenBank (accession number NC_024711.1) in the faeces of Chinese patients (Liang *et al*., 2016). Nevertheless, it was possible to find a conserved region of 78 bp, which was chosen to develop a qPCR assay for the molecular quantification of crAssphage.

### Validation of the crAssphage qPCR assay

**Figure 1:**
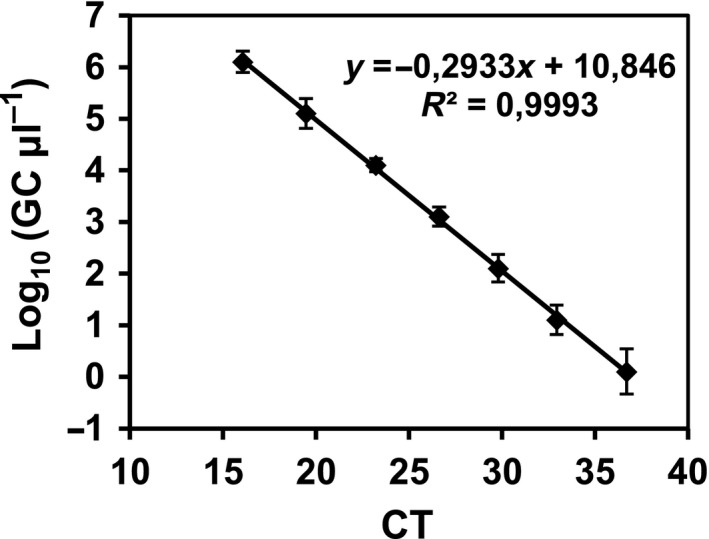
crAssphage qPCR assay. Standard curve obtained with pGEM plasmid containing the 1331‐bp sequence fragment obtained from a HM sewage sample DNA. CT, threshold cycle; GC, gene copies.

The standard curves of the crAssphage assay were reproducible. The average standard curve is shown in Fig. 1 with the corresponding equation. The qPCR assay was shown to have an efficiency of 100.4% and showed a limit of quantification (LOQ) of 1.27 gene copies (GC)/μl of the analysed sample (7 ul) of the qPCR mixture. The LOQ is defined as the last value of the standard curve that showed consistent and reproducible results and that is used to calculate the efficiency the qPCR assay.

### Quantification of crAssphage in sewage and wastewater samples

crAssphage was detected in the viral DNA and the total DNA fraction, although the concentration was around 1 log_10_ units lower than in total DNA fraction (data not shown). crAssphage has never been isolated, and we are not certain of their prevalence as a free virion in environmental settings and, therefore, we decided to use the total DNA fraction to analyse simultaneously free virions and prophages inserted within the *Bacteroides* genomes.

**Figure 2:**
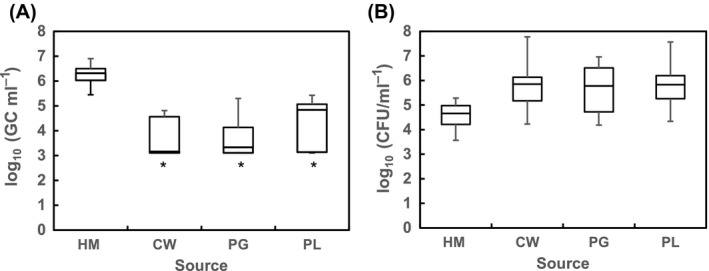
Densities (represented in box‐and‐whisker plot graphs) of (A) crAssphage (GC/ml) and (B) *Escherichia coli* (CFU/ml) in the different source samples: HM, human sewage (*n* = 23); PG, porcine wastewater (*n* = 15); CW, cow wastewater (*n* = 12); PL, poultry wastewater (*n* = 14). *, in these samples the lowest fence value in the graph corresponds to the limit of quantification of the qPCR as some of the samples were considered negative in the qPCR; therefore, the actual lowest value is expected to be lower than the plotted value.

High concentrations of crAssphage (GC/ml) were detected in the total DNA fraction of 100% of the 23 samples of raw municipal sewage tested (Fig. 2A), with values ranging from 5.4 to 6.9 log_10_ GC/ml and an average value of 6.24 (standard error (SE) = 0.1). These values are higher than those reported for other microbial source markers found in high numbers such as norovirus (da Silva *et al*., 2007), adenovirus and polyomavirus (Bofill‐Mas *et al*., 2006), human bifidobacteria (Gómez‐Doñate *et al*., 2012) and human Bacteroidetes (Gómez‐Doñate *et al*., 2016; Mayer *et al*., 2016).

However, crAssphage was also found in samples contaminated with faecal remains from certain animals, though in lower concentrations than in the human sources. Sixty‐one per cent of the samples (*n* = 41) collected from sources contaminated with faeces of different animals were positive for the presence of crAssphage. Logarithmic values presented as box‐and‐whisker plot graphs are shown in Fig. 2A. In this case, the values ranged from 3.2 to 4.8 log_10_ GC/ml in CW, 3.2 to 5.3 log_10_ GC/ml in PG and 3.2 to 5.4 in PL samples, with average values of 3.7 (SD = 0.75), 3.7 (SD = 0.67) and 4.1 (SD = 0.95) for the different animal wastewaters respectively. The concentration of the phage was significantly (*p* < 0.05) higher in human than in animal wastewaters.

The crAssphage sequence was originally identified from metagenomic analysis of human samples and was reported to be highly abundant in humans (Dutilh *et al*., 2014). To the best of our knowledge, there is only one published study of the prevalence of this phage in non‐human samples (Stachler and Bibby, 2014). Stachler and Bibby studied the presence of the phage in metagenomes from different animals including cow, pig and chicken, but the phage was not detected in any sample, except those from bats. In our study, crAssphage was detected in more than half of the animal samples analysed but in lower densities than in human samples. These differences between the results of the two studies may be due to two main reasons. First, the low number of samples analysed by Stachler and Bibby (*n* was between 1 and 4 for the genomes analysed); second, crAssphage may be not the dominant phage in animal samples and may not be detected in metagenomics analysis, but may be detected when targeting a specific sequence. In any case, the high abundance of this phage found in our study is important for developing an MST method, so that small amounts of faecal contamination can be detected. However, according to our results, the detection of its presence on its own may not be sufficient to track the origin of the contamination. Therefore, the use of the bacteriophage in combination with another faecal marker was studied.

### crAssphage and *Escherichia coli* for tracking human faecal contamination

**Figure 3:**
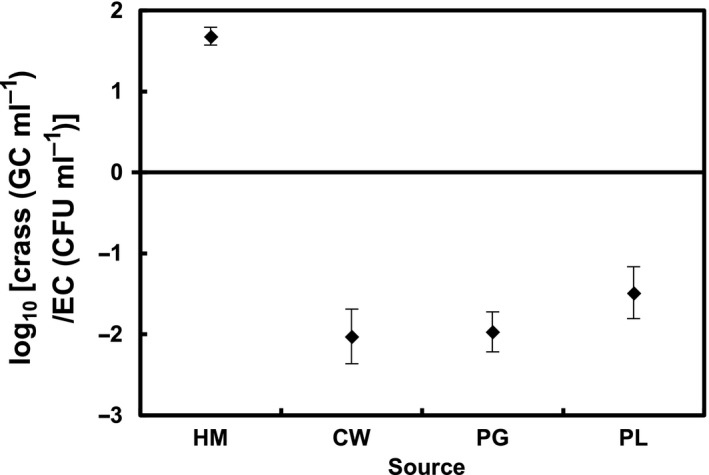
Observed log_10_ ratio [crAssphage (GC/ml)/*Escherichia coli* (CFU/ml)] for the different faecal pollution sources (mean ± standard error). crass: crAssphage; EC:* E. coli*.

As determined by the concentration of the universal indicator *E. coli*, the different samples tested had quite different concentrations of faecal residues with the highest values corresponding to the samples of animal origin (Fig. 2B). To examine the potential effect of the concentration of faecal remains in the samples, the crAssphage results were considered versus the abundance of *E. coli* by calculating the log_10_ value of the ratio between crAssphage (GC/ml) and *E. coli* (CFU/ml) (i.e. log_10_ [crAssphage (GC/ml)/*E. coli* (CFU/ml)]). The arithmetic means (± standard error) of the log_10_ values of these ratios are plotted in Fig. 3.

The log_10_ values of the ratios differed significantly (Kruskal–Wallis, *p *< 0.05) between human (ratio average = 1.7, min = 0.6 and max = 2.3) and non‐human samples (ratio average < −1.5, max = −0.9 and min = −4.7 for CW, max = −0.7 and min = −3.4 for PG, and max = 0.33 and min = −3.9 for PL), and therefore, it was possible to distinguish human from non‐human pollution source, based on the use of only two markers.

Field studies using most of the numerous chemical and microbiological methods available to track sources of faecal contamination have shown that the existing methods are insufficient and that different markers are needed (Blanch *et al*., 2004; Muniesa *et al*., 2012). Other authors have also indicated that the abundance ratio between a discriminating and non‐discriminating marker may be a useful tool. For example, in the late 1960s, Geldreich and Kenner (Geldreich and Kenner, 1969) proposed the ratio faecal coliforms/*Streptococcus fecalis*, and more recently, Sauer *et al*. (2011) discussed the usefulness of the abundance ratio human *Bacteroidales*/total *Bacteroidales* and Newton *et al*. (2011) proposed the use of the abundance ratio among Lachno2, a human‐associated phylotype within the *Lachnospiraceae* family and quantitative values of enterococci obtained by qPCR. And more recently, Muniesa and coworkers reported the usefulness of the ratio somatic coliphages/phages infecting *Bacteroides thetaiotaomicron* GA17 strain to predict human faecal pollution sources (Muniesa *et al*., 2012).

In conclusion, a methodology was developed to detect human faecal polluted water based on the quantification of just two markers: the recently reported crAssphage and the worldwide used indicator *E. coli*. Accordingly, crAssphage was present in some animal samples, but the use of the log_10_ ratio obtained among crAssphage/*E. coli* was suitable for the significant differentiation of human versus diverse animal faecal pollution sources. Furthermore, the observed numbers of crAssphage are amongst the highest reported for any human marker so far and therefore the proposed method besides being easy to perform, and robust could be used in highly diluted samples. These results are quite promising and make further research worthwhile to establish if different specific animal variants of crAssphage exist, and whether other genome regions of crAssphage are better suited to discriminating the animal source.

## Experimental procedures

### Wastewater samples

A total of 64 wastewater samples were collected from various sources. Twenty‐three samples were obtained from different urban wastewater treatment plants (WWTPs) (HM samples) serving populations ranging from 5000 to 384 000 inhabitants in Catalonia (NE Spain). A total of 14 samples of poultry slurry (PL samples) were collected from two poultry slaughter houses that each slay 60 000 animals per week. A total of 15 samples of pig slurry (PG samples) were obtained from four pig abattoirs that slay 12 500, 15 000, 15 000 and 5000 pigs per week. Finally, a total of 12 samples of cattle slurry (CW samples) were collected from four slaughterhouses butchering weekly between 250 and 2000 calves.

### Detection of *E. coli*

*Escherichia coli* was used as indicator of bacterial faecal pollution. *Escherichia coli* was enumerated by membrane filtration based on the ISO standard method 16649‐1:2001 with an initial resuscitation stage on MMGA (4 h at 37 °C) followed by incubation in chromogenic TBX agar at 44 °C (ISO, 2001).

### Total DNA extraction

Total DNA was isolated from 0.2 ml samples with the QIAamp DNA blood minikit (Qiagen GmbH, Hilden, Germany), following the manufacturer's instructions. The DNA was suspended in a final volume of 200 μl of elution buffer. The integrity of the genomic DNA extracted was evaluated by 0.8% agarose gel electrophoresis and ethidium bromide staining.

### Bacteriophage DNA extraction

The bacteriophage DNA fraction was extracted from 0.2 ml samples previously filtered through low‐protein‐binding 0.22 μm‐pore‐size membrane filters (Millex‐GP, Millipore) using the method described above.

### End‐point PCR assay

DNA was amplified by PCR from 10 HM samples using primers derived from the crAssphage sequence available in GenBank (accession number NC_024711.1) (Fig. S1) targeting a 1331 bp fragment (crAss1 5′‐CTGATAGTATGATTGGTAATG‐3′ and crAss2 5′‐AAGATAGTTGGAGAACTTAT‐3′) and sequenced using the ABI Prism BigDye 3.1 terminator cycle sequencing ready reaction kit (Life Technologies, Madrid, Spain).

### Quantitative PCR assay

A qPCR assay was developed based on the previously obtained consensus sequences from HM samples. crAss‐UP primer (5′‐AGGAGAAAGTGAACGTGGAAACA‐3′), crAss‐LP primer (5′‐TAAAGCTTAAAGTTGGTGCTCGTT‐3′) (which differed in a single base pair from the crAssphage sequence available at GenBank) and a Taqman probe with a 3′‐FAM carboxyfluorescein reporter and a 5′‐MGBNFQ (minor groove binding non‐fluorescent) quencher (FAM‐AGGATTTGGAGAAGGAA‐MGBNFQ) were designed using Primer Express 3.0.1 (Applied Biosystems) to amplify a 78‐bp fragment encoding the KP06_gp31 gene. The qPCR was performed using 7 μl of the DNA (total or phage DNA) extracted from each wastewater sample. The assay was performed under standard conditions as previously described (Gómez‐Doñate *et al*., 2012). All the samples were run in duplicate. Threshold cycle (Ct) data were expressed as the number of GC according to the values obtained with the standard for each qPCR reaction.

To generate standards for the qPCR assay, the 1331‐bp fragment was amplified from a HM sample. The amplified 1331‐bp fragment was cloned into the pGEM‐T Easy vector following the manufacturer's instructions (Promega Biotech Ibérica, Barcelona, Spain) and transformed by electroporation (2.5 kV, 25 F capacitance and 200 Ω resistance) into *E. coli* DH5α electrocompetent cells. The ampicillin‐resistant colonies containing the vector with the insert were selected, verified by PCR and used to purify the plasmid using a Qiagen Plasmid Midi purification kit (Qiagen, Valencia, CA). A NanoDrop ND‐1000 spectrophotometer (Thermoscientifics, Wilmington, DE) was used to evaluate the concentration and purity of the construct containing each band.

To calculate the number of gene copies (GC) in the prepared stock, the following equation was used as follows: [concentration of pGEM‐T Easy::insert (ng/μl)/molecular mass (ng/mol)] × 6.022 × 10^23^ molecules/mol = number of molecules of pGEM‐T Easy::insert/μl. Ten‐fold serial dilutions of the stock were performed with double‐distilled water and stored at −80 °C until used. The stocks were amplified in duplicate in five independent experiments, and the average of the Ct results was used to elaborate standard curves.

### Statistical analyses

The data corresponding to the different sample types are presented as box plots of multiple variables. Comparison of data from all types of samples was conducted using the Kruskal–Wallis test (statgraphics plus software (version 5.1)). For ratio calculations, the limit of detection was used for samples which were below the limit of detection.

## Conflict of Interest

None declared.

## Supporting information


**Fig. S1.** CrAssphage qPCR assay. Location of primers and probes designed for the end‐point and qPCR assay.
