# Supplementary figures and images for: Determination of crAssphage in water samples and applicability for tracking human faecal pollution
Source: Microb Biotechnol. 2017 Sep 19;10(6):1775–80. doi: 10.1111/1751-7915.12841 (PMC5658656; doi:10.1111/1751-7915.12841)

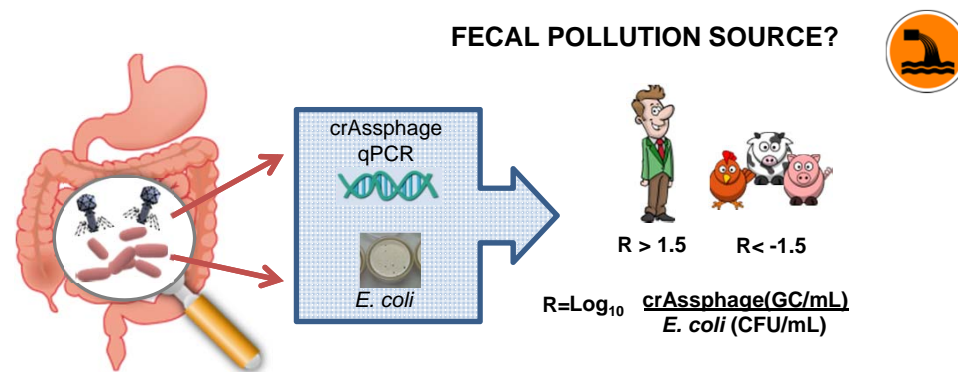

Supplement: Supplementary file 1 — Fig. S1. CrAssphage qPCR assay. Location of primers and probes designed for the end‐point and qPCR assay. [file MBT2-10-1775-s001.pdf]
